# Factors Associated with Exposure to Dietary Bisphenols in Adolescents

**DOI:** 10.3390/nu13051553

**Published:** 2021-05-05

**Authors:** Virginia Robles-Aguilera, Yolanda Gálvez-Ontiveros, Lourdes Rodrigo, Inmaculada Salcedo-Bellido, Margarita Aguilera, Alberto Zafra-Gómez, Celia Monteagudo, Ana Rivas

**Affiliations:** 1 Department of Nutrition and Food Science, University of Granada, Cartuja Campus, 18071 Granada, Spain; vraguilera@hotmail.com (V.R.-A.); yolandagalvez@ugr.es (Y.G.-O.); celiams@ugr.es (C.M.); amrivas@ugr.es (A.R.); 2 Instituto de Investigación Biosanitaria ibs-GRANADA, 18014 Granada, Spain; maguiler@ugr.es (M.A.); azafra@ugr.es (A.Z.-G.); 3 Department of Legal Medicine and Toxicology, University of Granada, 18071 Granada, Spain; lourdesr@ugr.es; 4 Department of Preventive Medicine and Public Health, University of Granada, Cartuja Campus, 18071 Granada, Spain; 5 Consortium for Biomedical Research in Epidemiology & Public Health (CIBER en Epidemiología y Salud Pública—CIBERESP), Monforte de Lemos 5, 2809 Madrid, Spain; 6 Department of Microbiology, Faculty of Pharmacy, University of Granada, Cartuja Campus, 18071 Granada, Spain; 7 Department of Analytical Chemistry, University of Granada, Fuentenueva Campus, 18071 Granada, Spain

**Keywords:** bisphenol A, bisphenol S, dietary exposure, adolescents, body mass index

## Abstract

Obesogenic endocrine-disrupting chemicals, such as bisphenol A (BPA) and its analogue bisphenol S (BPS), seem to play an important role in the development of obesity, although contradictory results have been reported. The aim of the present study was to conduct a gender analysis of the factors associated with exposure to dietary bisphenols in 585 Spanish adolescents. Dietary BPA and BPS exposure was assessed using a food frequency questionnaire. Foods and macronutrients accounting for more than 95% of energy intake were selected for analysis. Stepwise regression was used to estimate the foods that most contributed to dietary bisphenol exposure in the sample. Gender-related factors associated with greater dietary bisphenol exposure were evaluated using multivariate logistic regression models. Canned tuna was the main dietary source of BPA and BPS in both adolescent boys and girls. Overweight/obese girls showed a higher risk of high dietary exposure to BPA (odds ratio (OR): 3.38, 95% confidence interval (CI): 1.25–9.07) and total bisphenols (OR: 2.81, 95% CI: 1.03–7.67) in comparison with girls with a BMI lower than 25 kg/m^2^. Present results indicate a positive association of dietary exposure to both total bisphenols and BPA with being overweight/obese in adolescent girls.

## 1. Introduction

According to the World Health Organization, obesity is one of the most important current public health issues around the world [1]. Obesity is a complex condition with serious environmental, genetic, psychological, social, and economic dimensions. However, all of the causes and mechanisms involved in the development of obesity are not yet completely understood [2,3]. In addition, obesity prevalence in children and adolescents aged 2–18 years is rapidly growing [4], placing them at higher risk of adulthood obesity and of suffering metabolic disorders, cardiovascular diseases, and cancer at earlier ages [5,6]. Environmental factors have also been proposed as contributors to obesity, and there is a growing concern over obesogens. Obesogens are environmental chemicals with potential obesity-related endocrine-disrupting properties [7].

In this regard, bisphenol A (BPA) is an endocrine-disrupting chemical (EDC) suspected to have obesogenic properties [8]. BPA is a synthetic compound used in the lining of many food products, plastic bottles, and dental sealants. It is also used in thermal paper to give color [9]. In 2010, the use of BPA in baby products such as sippy cups, baby bottles, and infant formula packaging was banned in Europe and Canada because of its negative health effects [10,11]. In 2012, the US Food and Drug Administration also banned its use in baby products [12]. As a consequence, BPA started to be replaced by BPA analogues such as bisphenol S (BPS). This is used in thermal receipt paper, and BPS coatings are used in food and beverage cans [13]. A study conducted by Ye et al. (2015) investigated exposure trends for different bisphenols using adult urine samples. This study revealed that BPS exposure was on the rise, whilst BPA exposure was in decline, despite the fact that urine BPS concentrations were found to be lower than BPA concentrations [14]. Given that BPA and its analogues share the same basic chemical structure, there is also a risk of analogues acting as endocrine disruptors, a risk evidenced in in vitro and in vivo studies [15]. Bisphenols are now ubiquitous in the environment, with dietary exposure being one of the main routes through which humans are exposed to these compounds [16,17,18,19]. Food contamination with these chemicals typically occurs during food processing, packaging, transportation, and storage [20].

In vitro and in vivo studies have demonstrated the obesogenic role of bisphenols [21,22,23]. However, the limited number of epidemiological studies to have addressed the association between exposure to bisphenols and obesity have reported contradictory results [24,25,26,27]. A study published by Do et al. (2017) with 4733 adults aged between 18 and 79 years found higher levels of urinary BPA to be positively associated with obesity risk (odds ratio (OR): 1.54, 95% confidence interval (CI): 1.00–2.37) [28]. These outcomes are consistent with those reported by other large-scale cross-sectional studies conducted in the United States and China [28,29,30,31]. The National Health and Nutrition Examination Survey (NHANES), conducted between 2003 and 2006, included a number of cross-sectional studies designed to assess the health and nutritional status of adults and children in the United States. This national survey found that urinary BPA concentrations were positively associated with general and abdominal obesity. Specifically, relative to participants in the lowest BPA quartile, participants in the upper quartiles had 39–62% greater odds of presenting with abdominal obesity [29]. Another cross-sectional study using pooled data from NHANES (2003–2008) found that higher urinary BPA concentrations were positively associated with body mass index (BMI) and waist circumference (WC). These outcomes were found both in the studied population overall and when stratifying according to gender and race/ethnicity [30]. Wang and collaborators also found a positive association between urinary and serum BPA concentrations and obesity in 3390 Chinese adults aged 40 and above, alongside a positive association between urinary BPA and abdominal obesity [31]. Takeuchi and collaborators found higher serum BPA levels Japanese women with polycystic ovary syndrome, regardless of their obesity status, and in obese women who did not present with this syndrome [32]. Another study showed that urinary BPA concentrations were higher in obese women and in women aged ≥40 [33]. Lastly, Zhao and collaborators reported a statistically significant linear trend between fat mass and BPA exposure, as well as between serum leptin levels and BPA exposure, in 246 healthy premenopausal women [34].

Higher exposure to some obesogens has been reported in children and adolescents relative to adults. This is associated with differences pertaining to the diet, developmental and physiological factors, and toxicant metabolism [35]. Childhood and adolescence are the most critical developmental stages at which vulnerability to obesogens is elevated, with exposure to even low doses resulting in severe effects. In contrast, fully developed detoxification pathways and the blood–brain barrier offer a protective effect against obesogens in adults [36]. In addition, metabolic rate is higher at early developmental stages than it is in adults, and this promotes an obesogenic effect [37,38]. It has been reported that human exposure to EDCs during early life may disrupt neuroendocrine-mediated processes that are critical for growth, energy metabolism, appetite control, adipogenesis, and glucose–insulin regulation, thereby increasing the risk of childhood obesity [35]. Available epidemiological studies on the obesogenic effects of early-life exposure to BPA have reported discrepant results. Studies have shown associations between higher early-life BPA exposure and both increased and decreased adiposity or overweight/obesity risk [35,39,40], whereas others have failed to find any association [41,42]. In this regard, a recent meta-analysis performed by Kim and collaborators of data from different epidemiological studies suggested that associations found between BPA exposure and increased risk of obesity in children could be causal [43]. Another study analyzed NHANES (2013–2016) data to investigate relationships between BPA, BPS, and bisphenol F (BPF) urine concentration and body mass in a sample of children and adolescents aged 6 to 19 years [44]. This study reported that BPS exposure was moderately positively associated with higher standardized BMI (i.e., obesity and severe obesity). BPS exposure–obesity associations were more evident after log-transforming the variables as opposed to when using quartiles. The authors also found an association between BPS and BPF urine concentrations and central obesity. Lastly, they also found a positive association of BPF exposure with overweight classifications and overall BMI *z*-scores. In contrast, no significant association was found between BPA and any of the examined body mass outcomes. 

Although the specific periods during which children are more vulnerable to bisphenol exposure have not been fully identified, exposure during adolescence may be critical due to the dramatic changes that take place in relation to hormonal levels and body composition during this life stage. In this regard, the main aim of the present study was to identify the factors with the greatest influence on increasing bisphenol intake in 585 Spanish adolescents. The study sought to conduct a gender analysis, in accordance with previous studies which reported health-related gender differences following EDC exposure [35,45,46].

## 2. Materials and Methods

### 2.1. Study Population

The present sample formed part of a larger study population which included 708 high-school students from Talavera de la Reina (Toledo, Spain). Participants were recruited in 2017–2018 to participant in a research project carried out by the Carlos Health Institute (*Instituto de Salud Carlos*). Eligible participants met the following selection criteria: (1) aged between 12 and 16 years; (2) responded to the food frequency questionnaire (FFQ); (3) height and weight data available. Thus, a total of 585 participants (53.4% boys) were included in the study. Participants were excluded who had a diagnosed illness at the time of recruitment (to ensure the recruitment of only healthy subjects). All participants gave written informed consent and had parental permission to participate. The study was approved by the Ethics Committee of the University of Granada.

### 2.2. Data Collection

Data used in the present study were taken from a database which includes more than 200 variables. Anthropometric measures (height and weight), sociodemographic variables (gender, age, parent’s occupational ranking, and number of siblings), lifestyle variables (smoking habits, daily physical activity, and sedentary behavior engagement), and variables obtained from the FFQ were selected. Parent’s occupational ranking was determined from international standard occupational classifications [47]. Classifications were as follows: (1) high level, managers and professionals; (2) mid-level, technicians and associate professionals, clerical support workers, services and sales workers, skilled agricultural, forestry, and fishery workers; (3) low level, craft and related trades workers, plant and machine operators and assemblers, and elementary occupations.

Height and weight were measured by trained personnel using an electronic scale and a wall-mounted stadiometer, respectively. BMI was calculated as weight in kilograms divided by height squared in meters. Participants were classified as underweight, normal weight, overweight, and obese using standards proposed by the International Obesity Task Force described by Cole et al. (2000, 2007) [48,49]. BMI cut-points were calculated for children and adolescents aged 2–18 years old. Cut-points are specified at 6 month intervals up until 18 years old, at which point adult cut-points are used and obesity is defined as a BMI ≥ 30 kg/m^2^. 

The FFQ includes 96 items divided into the following 12 food groups: dairy products (*n* = 10), eggs, meat, and meat products (*n* = 7), fish (*n* = 3), vegetables (*n* = 15), fruit and dry fruit (*n* = 15), legumes (*n* = 4), cereals (*n* = 5), bakery products, pastries, and sweets (*n* = 10), fats (*n* = 5), nonalcoholic beverages (*n* = 9), alcoholic beverages (*n* = 4), and miscellaneous (*n* = 9). Consumption frequency was classified as never, 1–3 times/month, one time/week, 2–4 times/week, 5–6 times/week, one time/day, 2–3 times/day, 4–6 times/day, and more than six times/day. Information regarding the type of food packaging (plastic, glass, metal, or cardboard) was also recorded. This version of the FFQ was previously validated [50]. 

### 2.3. Bisphenol Concentrations in Food and Estimation of Dietary Exposure

Given that not all consumed food could be chemically analyzed, the foods most commonly consumed by the study population were evaluated. In this way, the main determinants of energy and macronutrient intake (based on FFQ responses) could be determined. Once the most consumed foods were selected, their bisphenol content was chemically analyzed. In order to assess bisphenol exposure, mean food consumption values (g/day) were multiplied by each food’s corresponding bisphenol content (ng/100g of food). This produced values of overall exposure to BPA and BPS (ng/day) [51].

Thus, a total of 82 of the 96 food items listed on the FFQ were selected and analyzed. Using stepwise regression, these foods were identified as the greatest contributors to energy and macronutrient intakes. Total energy intake (kcal/day), as well as intake of carbohydrates, lipids, and proteins (g/day), provided the dependent variables. The contribution of each food to overall energy and macronutrient intake (kcal or g/day, respectively) provided the covariates. Analysis was stopped once the point was reached at which the inclusion of a new food did not significantly improve the model (*p* > 0.05).

Mean consumption of these selected food items was calculated by multiplying consumption frequency (servings/day) by portion size (g). Methodology pertaining to sample analysis and determination of bisphenol levels in the selected foods was previously described [52,53]. Briefly, once an extract of the selected food items was obtained, bisphenol concentrations were analyzed using ultra-high-performance liquid chromatography–tandem mass spectrometry. A total of 52% of the samples had bisphenol levels greater than quantification levels. 

Daily dietary exposure to bisphenols was calculated for each participant by multiplying the daily food intake (g/day) of each food product by its corresponding bisphenol concentration (ng/g).

### 2.4. Statistical Analysis

Means and standard deviations (SD) were calculated for continuous variables, whilst the distribution of absolute and relative frequencies was calculated for categorical variables (BMI, smoking status, parent’s occupational ranking). Student’s *t*-test was used to evaluate the differences observed in continuous variables, whilst Pearson chi-square test was used for categorical variables, and Fisher’s exact test was used for cases when the expected frequency was lower than 0.05.

In order to select the food items which most strongly predicted bisphenol intake (BPA and BPS), stepwise regression (forward selection) was used. Analysis was halted when model improvements were no longer statistically significant (*p* > 0.05). Logistic regression models were used to identify factors with the greatest influence on total bisphenol content (BPA + BPS), as well as on BPA and BPS dietary exposure (third tercile), from produced ORs and 95% CIs. The dependent variables of total bisphenol (BPA + BPS), BPA, and BPS intake (ng/day) were categorized according to terciles and analyzed as dichotomous variables (first and second terciles vs. third tercile). The contribution of each food item to overall consumption (g/day) was entered as a predictive factor. The two grouped lowest terciles (first and second terciles) provided the reference category (lowest bisphenol intake), with greater bisphenol intake corresponding to the highest tercile. Gender, age, BMI, family size (large), smoking habits, and parental occupational ranking were included as factors. SPSS v.23 (version 23, IBM^®^ SPSS^®^ Statistics, Armonk, NY, USA) was used for all statistical analysis; significance was set at *p* < 0.05. 

## 3. Results

Table 1 presents the main characteristics of the sample. The mean age of participants was 15 years. Significant gender differences in weight and height emerged, with adolescent boys being taller and heavier than adolescent girls. In the present sample, 28% of adolescent boys and 23% of girls were overweight, whilst 19% of boys and 11% of adolescent girls were obese. In addition, the percentage of boys who had never smoked was higher than that of girls. No other gender-related differences were found.

Table 2 presents previously reported concentrations of bisphenol in foods [53]. The presence of bisphenols in nonpacked fruit and vegetables may be explained by the fact that wastewater is the primary source of bisphenols in the environment and it is reused for irrigation [54]. Moreover, contamination could potentially occur during primary production activities. Furthermore, the ubiquity of plastics could also be related to the unexpected presence of bisphenols in food. Daily food intake and average dietary exposure to bisphenols is presented in Table 2. Male adolescents showed significantly higher daily BPA dietary exposure than female adolescents due to their greater intake of pastry (730.5 vs. 534.8 ng/day, *p* = 0.03), pizza (177.8 vs. 137.2 ng/day, *p* < 0.01), and chicken (95.2 vs. 78.1 ng/g, *p* = 0.01). They also had greater BPS exposure associated with their greater consumption of Serrano ham (609.3 vs. 462.3 ng/day, *p* = 0.01), green pepper (1067.5 vs. 818.8 ng/day, *p* = 0.03), and cake (22.0 vs. 12.7 ng/day, *p* < 0.01).

Table 3 presents the food products that most contributed (95%) to dietary bisphenol exposure in the present study population. Canned tuna was the main contributor to BPA intake in both male and female adolescents, followed by pastry. The main contributor to BPS dietary intake was canned tuna, followed by salted snacks in boys and mushrooms in girls.

Table 4 presents the factors found in the present study to have the greatest influence on total bisphenol, BPA, and BPS intake, according to gender. The logistic regression model developed showed that adolescent boys were at a greater risk of high dietary BPS exposure than girls. In addition, age and BMI were influential factors when it came to higher dietary exposure to total bisphenols, as well as to BPA and BPS, independently, although these outcomes only emerged in girls. Girls aged under 14 years were at a greater risk than their older counterparts of having high dietary exposure (third tercile) to bisphenol overall, as well as BPS, independently. Overweight/obese girls were at a greater risk of having high dietary exposure to total bisphenols and BPA than girls with a BMI lower than 25 kg/m^2^. Lastly, boys who spent more time engaged in sedentary pursuits were at greater risk of being included in the third tercile with regard to bisphenol dietary exposure.

## 4. Discussion

The present results show positive associations of overall dietary exposure to bisphenol and BPA exposure, independently, with being overweight/obese within a sample of Spanish female adolescents. Nonetheless, no association was found between dietary BPS exposure and BMI. Given that adolescence represents a period of rapid growth and development, adolescents may be more vulnerable to the effects of environmental toxicants than adults [55]. It was assumed that this could result in an amplification of the adverse effects related with exposure to BPA analogues. In addition, many adolescents may have been exposed to BPA analogues, such as BPS, during the perinatal period and childhood. The tendency of BPS to bioaccumulate, combined with the constant and daily exposure of adolescents to it, may result in an exposure to BPS that is similar to that of BPA [12].

Findings of the present study are consistent with those published in JAMA by Trasande and collaborators who, using data from the National Health and Nutrition Examination Surveys (NHANES) (2003–2008), found a link between BPA urine concentrations and obesity in 2838 children and adolescents aged 6–19 years [56]. Similar findings were also reported by Amin and collaborators. These authors found BPA exposure to be associated with obesity and cardiometabolic risk factors in a study conducted with 132 Iranian children and adolescents aged 6–18 years [2]. Furthermore, the authors reported that children and adolescents in the third tercile of BPA exposure were at a 12.48 times higher risk of obesity (OR: 12.48, 95% CI: 3.36–46.39, *p*-value <0.001). A study conducted in children aged 6–11 using data from the Canadian Health Measures Survey (2007–2009) also found higher BPA concentrations in urine [57]. Moreover, a cross-sectional study in children from NHANES (2003–2008) reported a linear, positive, and significant association between urinary BPA concentrations and BMI within a representative sample of children analyzed via gender- and age-adjusted models. This association was stronger within a subgroup of non-Hispanic white boys, with outcomes failing to reach statistical significance in the other examined ethnic subgroups [58]. These outcomes are consistent with those reported by Trasande et al. (2012) using data from NHANES 2003–2008. These authors found that children with urinary BPA profiles corresponding to second, third, and fourth quartiles had a higher prevalence of obesity (second quartile: 20.1%, 95% CI: 14.5–25.6%; third quartile: 19.0%, 95% CI: 13.7–24.2%; fourth quartile: 22.3%, 95% CI: 16.6–27.9%) than those with profiles corresponding to the lowest quartile (10.3%, 95% CI: 16.6–27.9%). Stratified analysis showed that this significant positive only emerged in white participants but not in participants of other ethnicities [56]. Lastly, a study conducted by Eng et al. (2013), also using NHANES data (2003–2010), reported that children in the three upper BPA quartiles had higher odds of presenting with obesity and higher waist circumference/height ratios than children in the first quartile [59]. A study conducted in China showed that girls aged 9–12 years with raised BPA levels (≥2 μg/L) appeared to be at a higher risk of being overweight (OR: 2.32, 95% CI: 1.15–4.65) [45]. Another study conducted in China also found a significant positive linear correlation between BPA urine concentration and BMI in children aged 8–15 years, alongside higher urinary BPA levels in obese children [60]. However, studies conducted in India and the US found a negative association between BPA and obesity in children. This contradictory finding may be explained by the small sample size available in these studies, meaning that their outcomes should be considered with caution [61,62].

Interestingly, the present study found a positive association between BMI and dietary exposure to both total bisphenols and BPA in girls but not in boys. This is in accordance with other epidemiological studies which also reported sex-related differences [45,56,58,60]. In this regard, environmental factors have been reported to generally have a greater impact on weight in girls than in boys [63]. Three previously conducted scientific works found an association between prenatal BPA exposure and lower BMI, with this association being stronger in girls [40,64,65]. Li et al. (2013) reported an association between high urine BPA levels and overweight status within girls aged 9–12, with no associations being found in boys [45]. Nonetheless, another study failed to find sex-related differences [56]. A recent meta-analysis of data collected in adults and children reported a dose–response analysis of different studies. This analysis found that a 1 ng/mL increase in BPA levels increased the risk of obesity by around 11% [66]. 

Gender-related differences in associations between BPA exposure and obesity have also been reported in animal models. This may be explained by differences in BPA metabolism and in the expression of the estrogenic receptor, as well as by gender-related dietary BPA exposure and energy expenditure [67,68,69,70]. Some studies reported that sex-related differences in hormone profiles may result in different adverse responses to BPA exposure [15,60,71]. BPA can selectively modulate estrogenic receptors and, thus, harmful or adverse effects related to BPA exposure will depend on the tissue [72]. In this regard, Xu et al. (2011) reported that BPA may act as an estrogenic agonist in males with low estrogen levels and an estrogenic antagonist in females with high endogenous estrogen levels [73]. The biological plausibility of this warrants further research in order to determine the sex-specific health differences associated with BPA exposure.

The time spent engaged in sedentary activities was a significant factor predicting high dietary exposure to bisphenols. Greater time spent engaged in sedentary activities increased the risk of having a high total bisphenol intake but only in boys. Although some authors reported similar findings, it is currently unclear why these lifestyle factors are associated with bisphenol exposure [74,75,76].

In the present study, no association was found between dietary BPS exposure and BMI, although previous in vivo and in vitro studies suggested that BPS exhibits similar obesogenic activity to BPA [15,77]. Liu et al. (2017, 2019) found a positive and statistically significant association between BPA exposure and obesity in boys, but not girls, from a representative sample of adolescents. These authors did not find a significant association between BPS exposure and obesity, with this outcome being consistent with present findings [46,78].

Observed gender differences in dietary bisphenol exposure may be explained by their different food preferences, as revealed in Table 2. Nevertheless, boys were only found to have a higher risk of dietary BPS exposure, with BPA exposure risk being similar between genders. Previous studies failed to find gender differences with regard to BPA exposure, although outcomes pertaining to BPS exposure were more equivocal [74,79]. In this sense, Chen et al. (2018) did not find significant sex-related differences in BPA and BPS urinary levels between 122 boys and 91 girls aged 3–11 years [79]. Lehmler et al. (2018) also reported no sex-related differences in BPA exposure in a sample of 429 boys and 439 girls aged 6–11 years from NHANES 2013–2014, although they did find higher BPS urinary levels in girls relative to boys [74]. However, given that both of these studies determined urinary BPA and BPS levels, their results cannot be considered in line with those of the present study. Given that diet is an important source of human exposure to bisphenols [16,17,18,19], gender-related differences found in BPS exposure could be due to the fact that boys consume more foods with high BPS levels than girls. In fact, boys consumed more Serrano ham, green pepper, and cake than girls, as can be observed in Table 2. Age-related differences in dietary exposure were also found, with girls younger than 14 years being at a higher risk of having high dietary exposure to total bisphenols and BPS. These results may be explained by the increased energy intake in girls younger than 14 years increasing exposure to bisphenols in the diet. Energy intake in girls older than 14 years decreases as a consequence of the termination of the growing process which occurs at around 16 years old [80].

### Strengths and Limitations

To the best of our knowledge, this is the first Spanish study to investigate the factors associated with dietary bisphenol exposure in adolescents. Advantages of the present study include a large sample size, use of trained personnel to obtain anthropometric measurements, and use of a previously validated questionnaire.

The present study also has some limitations. Firstly, due to the cross-sectional nature of the data, temporal relationships could not be established. Nonetheless, findings can serve as a basis for future research. Secondly, multiple sources of BPA exposure exist in adolescents outside of the diet which were not considered in the present study because biological samples were not collected. However, the diet is considered to be one of the main sources of exposure [16,17,18,19]. Lastly, obesity is a complex health issue whose definition is still a subject of controversy.

## 5. Conclusions

The current study is the first to report the association among factors that influence dietary bisphenol exposure in adolescents. The results indicate differences in dietary bisphenol exposure between girls and boys. Nevertheless, a higher risk of dietary exposure among boys was found only for BPS but not for BPA. 

Whilst the conventional model shows obesity/overweight to be caused by an energy imbalance in which energy intake exceeds energy expenditure, the role of environmental obesogens such as bisphenols should not be ignored. Furthermore, the positive association found between total bisphenols and BPA dietary exposure and BMI in the present sample of adolescents may suggest that exposure to even relatively low levels could be related to health issues. Moreover, the results showed gender-related differences in the association between BPA exposure and obesity. Nonetheless, further epidemiological and toxicological studies are required to investigate whether bisphenol exposure increases obesity risk in adolescents considering gender-related differences, ideally using a longitudinal design and including measurements of bisphenols in biological samples. 

## Figures and Tables

**Table 1:** Characteristics of the overall sample (*n* = 585).

	Boys (*n* = 313)	Girls (*n* = 272)	*p*-Value
Age (years), mean (SD)	15.4 (2.2)	15.2 (2.3)	0.44 ^a^
Weight (kg), mean (SD)	62.9 (16.8)	57.1 (12.2)	**<0.01 ^a^**
Height (m), mean (SD)	1.7 (0.1)	1.6 (0.1)	**<0.01 ^a^**
BMI (kg/m^2^), mean (SD)	22.5 (4.7)	22.5 (4.2)	0.88 ^a^
BMI, *n* (%)			0.52 ^b^
Underweight	8 (5.6)	3 (2.6)	
Normal weight	88 (61.5)	77 (67.5)	
Overweight	28 (19.6)	23 (20.2)	
Obesity	19 (13.3)	11 (9.7)	
Number of siblings, mean (SD)	1.2 (0.9)	1.2 (0.9)	0.99 ^a^
Smoking status, *n* (%)			**0.04 ^c^**
Never	231 (73.8)	182 (66.9)	
Former	28 (8.9)	42 (15.4)	
Current	54 (17.3)	48 (17.7)	
Number of cigarettes day, mean (SD)	1.0 (2.9)	1.1 (3.1)	0.65 ^a^
Father’s occupational ranking, *n* (%)			0.83 ^c^
Low qualifications	127 (45.7)	100 (43.3)	
Medium qualifications	132 (47.5)	113 (48.9)	
High qualifications	19 (6.8)	18 (7.8)	
Mother’s occupational ranking, *n* (%)			0.701 ^c^
Low qualifications	223 (76.6)	186 (76.2)	
Medium qualifications	32 (11.0)	23 (9.4)	
High qualifications	36 (12.4)	35 (14.3)	
Physical activity, mean (SD):			
Sedentary activities (hours/day)	8.98 (2.57)	9.14 (2.22)	0.210 ^a^
Physical activities (hours/day)	0.88 (0.57)	0.72 (0.43)	0.006 ^a^

SD: standard deviation; BMI: body mass index. *p*-Values <0.05 are highlighted in bold. ^a^ Student’s *t*-test; ^b^ Fisher exact test; ^c^ chi-square.

**Table 2:** Dietary intake of bisphenols (ng/day) according to gender.

	Boys					Girls			
Food	*n*	Packaging	Bisphenol Concentration, ng/g (SD)	Average Food Intake, g/day (SD)	Bisphenol Intake (ng/day), Mean (SD)	*n*	Average Food Intake, g/day (SD)	Bisphenol Intake (ng/day), Mean (SD)	*p*-Value ^a^
**Bisphenol A**									
Pastry	227	Plastic	41.5 (4.3)	17.6 (24.8)	730.5 (1030.6)	201	12.9 (21.1)	534.8 (874.1)	**0.03**
Pineapple	170	Plastic	11.3 (4.6)	12.2 (13.9)	137.5 (156.8)	130	13.3 (14.5)	150.7 (163.8)	0.48
Semi-cured cheese	182	Plastic	2.0 (0.3)	22.2 (27.7)	44.4 (55.5)	168	17.6 (23.7)	35.2 (47.4)	0.10
Canned tuna	166	Can	409.0 (23.2)	9.8 (10.0)	4027.2 (4106.5)	128	8.4 (9.2)	3418.9 (3778.6)	0.19
Pizza	285	Plastic	4.3 (1.8)	41.3 (44.3)	177.8 (190.8)	253	31.9 (30.7)	137.2 (132.2)	**<0.01**
Apple, pear	258	Not packaged, plastic	3.7 (2.0)	82.1 (93.8)	303.8 (346.9)	209	68.8 (80.4)	254.5 (297.7)	0.10
Sliced bread	237	Plastic	1.20 (0.3)	22.4 (24.2)	26.9 (28.9)	216	25.9 (25.4)	31.1 (30.4)	0.14
Chicken	294	Plastic and porex tray	2.1 (0.2)	45.3 (46.7)	95.2 (98.2)	248	37.2 (28.9)	78.1 (60.6)	**0.01**
Serrano ham	269	Plastic	17.3 (2.4)	15.5 (17.1)	268.2 (296.1)	239	14.1 (22.4)	243.5 (387.8)	0.42
Melon	195	Plastic	7.86 (3.7)	50.6 (79.1)	397.6 (621.8)	156	50.4 (77.9)	395.7 (611.9)	0.98
Canned corn	100	Can	42.7 (4.9)	12.4 (13.7)	529.1 (584.7)	82	10.1 (10.7)	430.6 (455.9)	0.20
Salted snacks	257	Plastic	25.45 (23.54)	11.1 (14.8)	282.1 (375.4)	233	9.4 (11.2)	238.8 (284.6)	0.15
Ham	221	Plastic	6.6 (3.4)	12.0 (14.5)	79.4 (95.4)	177	11.6 (13.5)	76.5 (89.3)	0.76
**Bisphenol S**									
Serrano ham	269	Plastic	39.3 (21.3)	15.5 (17.1)	609.3 (672.7)	235	11.8 (13.8)	462.3 (541.6)	**0.01**
Melon	194	Plastic	4.22 (0.67)	48.9 (75.8)	206.4 (319.8)	154	46.1 (68.9)	194.7 (290.7)	0.72
Carrot and pumpkin	178	Plastic	11.5 (5.3)	26.2 (32.5)	301 (385.4)	153	23.2 (21.0)	267.4 (241.8)	0.34
Mushroom	142	Plastic	16 (6.9)	49.0 (60.1)	784.2 (961.5)	140	43.8 (70.7)	700.3 (1130.5)	0.50
Green pepper	148	Plastic	27.5 (6.3)	38.8 (36.9)	1067.5 (1015.5)	133	29.8 (30.8)	818.8 (847.2)	**0.03**
Salted snacks	257	Plastic	132.1 (21.2)	11.1 (14.8)	1464.5 (1948.3)	233	9.4 (11.2)	1239.8 (1477.2)	0.15
Canned tuna	167	Can	187.8 (15.2)	10.1 (10.5)	1894.3 (1968.4)	130	9.0 (10.5)	1690.2 (1974.1)	0.38
Rice	295	Plastic	3.3 (1.4)	18.7 (23.0)	61.5 (75.9)	251	15.9 (18.9)	52.3 (62.4)	0.11
Cake	213	Not packaged	1.7 (0.7)	13.0 (18.7)	22.0 (31.8)	181	7.5 (8.1)	12.7 (13.7)	**<0.01**
Tomato	198	Not packaged and plastic	15.3 (14.9)	66.7 (72.8)	1021.0 (1113.6)	176	68.6 (74.2)	1049.7 (1134.8)	0.81
Semi-cured cheese	183	Plastic	5.3 (0.50)	23.7 (34.5)	125.7 (182.6)	172	23.6 (45.6)	125.1 (241.5)	0.98
Apple, pear	259	Not packaged, plastic	8.8 (3.80)	86.4 (116.5)	760.6 (1025.6)	212	84.8 (155.9)	746.1 (1372.3)	0.90
Ham	221	Plastic	5.43 (3.34)	12.0 (14.5)	65.3 (78.5)	177	11.6 (13.5)	62.9 (73.4)	0.76
Pineapple	171	Plastic	44.3 (2.8)	13.7 (20.3)	606.8 (898.7)	130	13.3 (14.5)	590.7 (642.2)	0.86
Olives	195	Plastic	19.4 (15.3)	12.9 (17.5)	249.4 (339.6)	173	10.8 (15.5)	210.5 (300.1)	0.25

SD: standard deviation. *p*-Values < 0.05 are highlighted in bold. ^a^ Student’s *t*-test.

**Table 3:** Food products that contribute to more than 95% of dietary bisphenol exposure according to gender (stepwise regression).

	Bisphenol A	*R^2^* ^a^	Bisphenol S	*R^2^* ^a^
**Boys**	Canned tuna	0.86	Canned tuna	0.35
	Pastry	0.95	Salted snacks	0.58
			Tomato	0.76
			Apple and pear	0.85
			Mushroom	0.91
			Green pepper	0.95
**Girls**	Canned tuna	0.88	Canned tuna	0.45
	Pastry	0.95	Mushroom	0.57
			Salted snacks	0.69
			Apple and pear	0.80
			Tomato	0.89
			Green pepper	0.94
			Pineapple	0.97

^a^ *R^2^*: determination coefficient as a proxy of model goodness of fit (range 0–1; *R^2^* of 1 indicates that regression predictions perfectly fit the data).

**Table 4:** The influence of factors on risk of having a high total intake (third tercile) of total bisphenols, bisphenol A, and bisphenol S, according to gender.

		Total Bisphenols				Bisphenol A				Bisphenol S					
Factor	Categories	OR	95% CI			OR	95% CI			OR	95% CI				
Gender	Ref. Girls														
	Boys	1.37	0.97–1.94			1.38	0.98–1.95			**1.45**	**1.02–2.04**				
		**Boys**		**Girls**		**Boys**		**Girls**		**Boys**			**Girls**		
		**OR**	**95% CI**	**OR**	**95% CI**	**OR**	**95% CI**	**OR**	**95% CI**	**OR**	**95% CI**		**OR**	**95% CI**	
Age	Ref. Age >14 years														
	Age ≤14 years	0.83	0.37–1.84	**4.77**	**1.57–14.55**	1.24	0.56–2.77	2.26	0.78–6.60	1.02	0.46–2.26		**4.24**	**1.44–12.48**	
BMI	Ref. BMI <25 kg/m^2^														
	BMI ≥25 kg/m^2^	0.97	0.43–2.21	**2.81**	**1.03–7.67**	1.02	0.45–2.32	**3.38**	**1.25–9.07**	1.40	0.62–3.15		2.36	0.86–6.44	
Large family	Ref. Yes														
	No	0.67	0.30–1.50	1.11	0.39–3.18	0.58	0.26–1.30	1.27	0.45–3.58	0.65	0.29–1.44		0.86	0.31–2.40	
Smoking status	Ref. Never														
	Former	1.36	0.45–4.11	1.51	0.36–6.39	1.14	0.36–3.57	1.24	0.31–5.04	1.43	0.47–4.34		1.73	0.44–6.77	
	Current	0.88	0.27–2.83	1.61	0.46–5.60	1.05	0.32–3.41	1.26	0.38–4.21	1.06	0.34–3.33		1.14	0.34–3.87	
Father’s occupational ranking	Ref. Medium–high qualifications														
	Low qualifications	0.99	0.46–2.12	1.09	0.43–2.76	1.29	0.60–2.79	0.71	0.28–1.80	0.69	0.32–1.47		0.64	0.25–1.63	
Mother’s occupational ranking	Ref. Medium–high qualifications														
	Low qualifications	0.70	0.29–1.68	1.05	0.29–3.80	1.14	0.46–2.83	1.25	0.35–4.45	0.95	0.39–2.31		1.11	0.32–3.89	
Physical activity	Sedentary activities (hours/day)	**1.10**	**1.07–1.20**	1.03	0.92–1.15	1.05	0.96–1.15	1.04	0.93–1.17	1.07	0.98–1.17		1.09	0.97–1.22	
	Physical activities (hours/day)	0.84	0.50–1.38	1.37	0.61–3.07	0.78	0.47–1.21	1.18	0.52–2.66	0.87	0.53–1.43		1.13	0.49–2.58	

BMI: body mass index; Ref.: reference category; OR: odds ratio; 95% CI: confidence interval. *p*-Values <0.05 are highlighted in bold.

## Data Availability

The data that support the findings of this study are available on request from the corresponding author.
